# Tuina therapy alleviates knee osteoarthritis by modulating PI3K/AKT/mTOR-mediated autophagy: an integrated machine learning and *in vivo* rat study

**DOI:** 10.3389/fimmu.2025.1635818

**Published:** 2025-10-01

**Authors:** Zhen Wang, Chi Zhao, Mengmeng Li, Lili Zhang, Jieyao Diao, Yiming Wu, Tao Yang, Mingwei Shi, Yang Lei, Yu Wang, Miaoxiu Li, Yanqin Bian, Yunfeng Zhou, Hui Xu

**Affiliations:** ^1^ College of Acupuncture and Massage, Henan University of Chinese Medicine, Zhengzhou, China; ^2^ Acupuncture and Massage Department, The Third Affiliated Hospital of Henan University of Chinese Medicine, Zhengzhou, China; ^3^ College of Computer Science, Xidian University, Xian, China; ^4^ College of Acupuncture and Massage, Shanghai University of Chinese Medicine, Shanghai, China; ^5^ Orthopedic Research Laboratory, University of California, Davis, Davis, CA, United States

**Keywords:** PI3K/Akt/mTOR pathway, autophagy, Tuina, knee osteoarthritis, 740 Y-P, LY294002

## Abstract

**Background:**

Previous studies suggest that Tuina therapy may alleviate knee osteoarthritis (KOA) by modulating the PI3K/AKT/mTOR signaling pathway and autophagy. However, these findings require validation. This study investigated the effect of Tuina monotherapy and Tuina therapy in combination with either a PI3K/AKT/mTOR pathway inhibitor or agonist to investigate whether Tuina therapy alleviates KOA progression by targeting a PI3K/AKT/mTOR pathway to regulate chondrocyte autophagy.

**Methods:**

A KOA rat model was established by intra-articular injection of L-cysteine-activated papain solution into the right knee. Rats were randomized to seven groups: Control, Model, LY294002 (PI3K/AKT/mTOR inhibitor), 740 Y-P (PI3K/AKT/mTOR agonist), Tuina, Tuina+LY294002, and Tuina+740 Y-P. The paw withdrawal threshold, knee swelling, and passive range of motion were used as behavioral outcomes. Cartilage degeneration was evaluated using hematoxylin and eosin and Safranin O-Fast Green staining. Chondrocyte ultrastructure and autophagy were observed using transmission electron microscopy. mRNA and protein expression of the PI3K/AKT/mTOR pathway and its downstream biomarkers were quantified using quantitative real-time polymerase chain reaction (RT-qPCR), immunohistochemistry, and western blotting. A secondary analysis was conducted using a support vector machine (SVM) algorithm to predict therapeutic effects and synergistic correlations between indicators.

**Results:**

Tuina reduced pain and improved function in KOA model rats, reduced cartilage and chondrocyte damage, increased the cartilage area, and reduced the level of autophagy. Tuina downregulated ATG5, ATG7, ULK1, Beclin-1, LC3II/I and upregulated PI3K, AKT, mTOR, and P62 in cartilage. Compared with the Tuina monotherapy group, the Tuina+LY294002 group had greater pain, joint dysfunction, cartilage degeneration, reduced cartilage area, elevated autophagy, and reduced PI3K/AKT/mTOR pathway activity, whereas Tuina+740 Y-P had the opposite effect. Machine learning validation through SVM achieved 97.62% predictive accuracy. Autophagy was strongly correlated with the PI3K/AKT/mTOR signaling pathway, cartilage degeneration, and behavioral assessment. 740 Y-P enhanced the effect of Tuina therapy, whereas LY294002 attenuated its effect.

**Conclusion:**

Tuina therapy mitigates cartilage degradation and delays KOA progression by activating the PI3K/AKT/mTOR pathway to inhibit chondrocyte autophagy. This study provides insights into the mechanisms through which Tuina exerts its therapeutic effect and highlights its potential as a non-pharmacological intervention for KOA.

## Introduction

Knee osteoarthritis (KOA), the most prevalent joint disorder, affects approximately 365 million adults worldwide (1). Risk factors include advancing age, obesity, joint injury, and genetic predisposition (2–4). As a leading contributor to pain and disability, KOA manifests as progressive articular cartilage degradation, chronic synovial inflammation, and pathological subchondral bone remodeling (5). Although pharmacological therapy is the first-line intervention, chronic use is associated with gastrointestinal, cardiovascular, and renal complications (6, 7). Surgical options, including arthroscopic procedures and knee arthroplasty, offer substantial therapeutic benefits and immediate symptom relief, but are associated with the risk of surgical site infection and thromboembolic events (8). In addition, current treatment does not address the underlying molecular mechanisms driving disease progression. Therefore, novel therapeutic targets and non-pharmacological strategies that modify disease progression are needed.

Tuina therapy, a traditional Chinese manual treatment grounded in meridian theory and biomechanical principles, is recognized as a non-pharmacological intervention for KOA, and is recognized for its multi-target regulatory effects, clinical effectiveness, and favorable safety profile (9, 10). Functionally, Tuina application facilitates meridian unblocking, regulates qi and blood circulation, enhances joint mobility, corrects abnormal stress distribution, and restores biomechanical equilibrium. Tuina has been shown to raise the pressure pain threshold, alleviate symptoms, and improve functional recovery (11–13). However, the precise molecular mechanisms underlying the therapeutic effects of Tuina on KOA pathophysiology have not been fully elucidated; therefore further research is warranted.

Dysregulated chondrocyte autophagy, a critical cellular homeostasis mechanism, has been implicated in the pathogenesis of KOA (14). Impaired autophagy exacerbates cartilage degeneration by promoting extracellular matrix catabolism and chondrocyte apoptosis, and its modulation is a potential therapeutic target (15). Multiple signaling pathways, including PI3K/AKT/mTOR, AMPK/mTOR, MAPK/NF-κB, and ERK1/2, play critical roles in regulating autophagy in chondrocytes (16, 17). Among these, the PI3K/AKT/mTOR pathway serves as a central regulator of autophagy and affects chondrocyte survival and cartilage tissue integrity in models of KOA. Once activated, this pathway suppresses the expression of several autophagy-related proteins such as ULK1, LC3, Beclin-1, which are essential for autophagosome formation. The PI3K/AKT/mTOR pathway inhibits chondrocyte autophagy through a multi-layered regulatory network, thereby contributing to the progression of KOA (18, 19).

Preliminary research from observational studies suggests that Tuina therapy may exert its therapeutic effects through PI3K/AKT/mTOR-mediated suppression of excessive chondrocyte autophagy (20). However, the causal relationships between pathway activation, autophagy modulation, and cartilage preservation have not been validated experimentally. As a classic competitive phosphoinositide 3-kinase (PI3K) inhibitor, LY294002 specifically binds to the ATP-binding catalytic domain of PI3K, thereby inhibiting the activation of the downstream AKT/mTOR pathway. It has been widely used to confirm the involvement of the PI3K/AKT/mTOR pathway in cartilage degeneration in KOA (21). Conversely, 740 Y-P is a cell-permeable PI3K agonist that activates this pathway by binding to the SH2 domain of the regulatory subunit p85 of PI3K, mimicking physiologically phosphorylated tyrosine-mediated activation (22). Applying LY294002 and 740 Y-P, enables the effects of Tuina therapy on KOA to be observed when the PI3K/AKT/mTOR signaling pathway is inhibited or activated, respectively. This approach helps explore whether Tuina therapy directly targets the PI3K/AKT/mTOR signaling pathway to suppress pathological autophagy, and whether combining massage with pathway-specific agonists or inhibitors enhances or diminishes its therapeutic outcomes. Understanding the mechanisms underlying the effects of Tuina therapy enables optimization of its clinical application.

Although animal experiments provide valuable insights into the mechanisms of disease intervention and remission, they have methodological limitations. Conventional experimental designs focus on isolated outcome measurement, and do not capture the holistic regulatory dynamics and interdependencies among biomarkers (23–25). This prevents comprehensive evaluation of the system, thereby limiting understanding of the therapeutic networks underlying the intervention effects and limiting the generalizability of the research findings.

Owing to its advanced data processing and pattern recognition capabilities, machine learning (ML) offers transformative potential for predictive analysis of complex biological datasets (26). Specifically, support vector machine (SVM) models demonstrate unique advantages in simultaneously processing multidimensional experimental parameters, effectively identifying intricate intervariable relationships that are often overlooked in traditional animal studies. Through kernel-based pattern recognition, SVM facilitates holistic interpretation of cross-dimensional biomarker interactions and therapeutic outcomes (27). This framework not only elucidates synergistic interaction networks among biological indicators but also enables predictive modeling of comprehensive therapeutic effects for intervention strategies (28, 29). The use of such computational approaches enhances understanding of the complex pathophysiological mechanisms and enables optimization of its therapeutic application.

This study used an interdisciplinary framework integrating *in vivo* rat experiments and ML to investigate the role of Tuina therapy in modulating the PI3K/AKT/mTOR-autophagy axis within a rat model of KOA. This study had two components: (1) conducting combined interventions using Tuina therapy with either the PI3K/AKT/mTOR pathway inhibitor LY294002 or agonist 740 Y-P to confirm the involvement of the PI3K/AKT/mTOR pathway; and (2) a support vector machine (SVM) model for secondary multimodal integration of experimental data, enabling predictive analysis of biomarker correlations and comprehensive efficacy evaluation across intervention modalities to validate and complement findings from the *in vivo* experiments. Our findings provide mechanistic insights into Tuina’s mechanism of action and support its potential as a non-invasive, autophagy-targeting therapy for KOA management.

## Materials and methods

**Figure 1:**
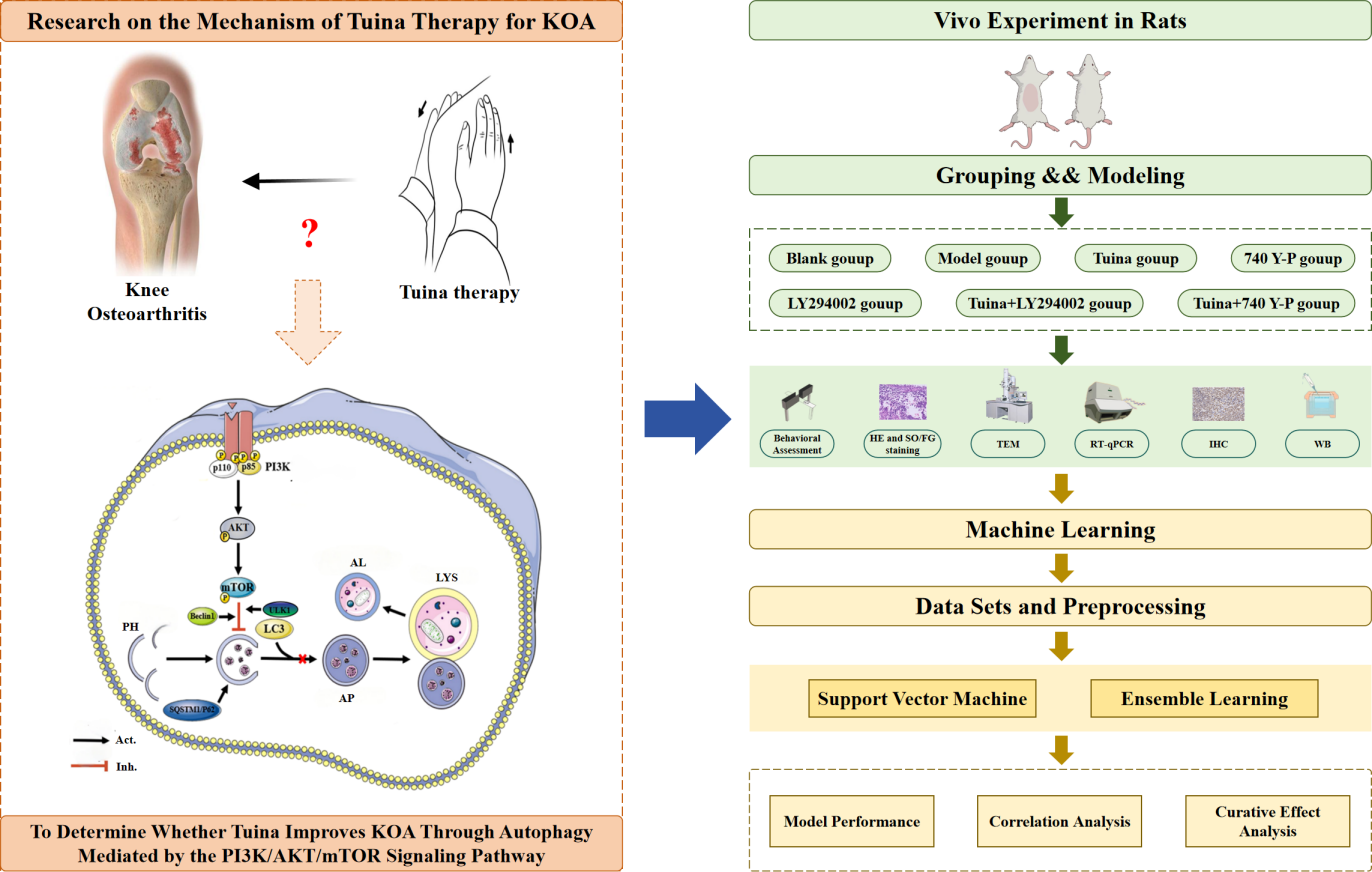
Overview technical route of the study design. KOA, knee osteoarthritis; PI3K, Phosphatidylinositol 3-kinase; AKT, Protein kinase B; mTOR, Mechanistic target of rapamycin; PH, Phagophore; AP, Autophagosome; LYS, Lysosome; AL, Autolysosome; HE, Hematoxylin and eosin; SO/FG, Safranin O/Fast Green; TEM, Transmission electron microscopy; RT-qPCR, Real-time quantitative polymerase chain reaction; IHC, Immunohistochemistry; WB, Western blot.

The study design is summarized in **Figure 1**. This study had two components: first, therapeutic efficacy was systematically evaluated through behavioral assessments including the paw withdrawal threshold (PWT), knee joint swelling (KJS), and passive range of motion (PROM). Histopathological evaluation of cartilage damage and degeneration was performed using hematoxylin and eosin (HE) and Safranin O/Fast Green (SO/FG) staining. Autophagy levels were quantified via transmission electron microscopy (TEM), and the expression levels of PI3K/AKT/mTOR signaling pathway biomarkers and downstream autophagy-related markers were measured using reverse transcription-quantitative PCR (RT-qPCR), western blot (WB), and immunohistochemical analyses. ML was then used to conduct further in-depth analyses and verify the experimental data, and the SVM model was used to predict the therapeutic effect of indicators and the correlation of their effects.

### *In vivo* experiment in rats

#### Experimental animals and groups

A total of 91 male Sprague-Dawley rats (weight, 280–320 g; age, 8–9 weeks) obtained from Beijing Vital River Laboratory Animal Technology Co., Ltd. (SCxK [Jing] 2021-0006) were housed individually under controlled conditions (20 °C ± 2 °C; 40–50% humidity) with 12-h light cycles and ad libitum access to standard chow and water. All procedures were approved by the Animal Experimental Ethics Committee of Henan University of Chinese Medicine (IACUC-202410026). The instrument and reagent specifications are provided in **Supplementary Table S1**.

After a 1-week acclimation period, the rats were randomly allocated to the blank (n=13) or model establishment (n = 78) group using a random number table. After model verification (1 rat per group), the remaining KOA rats were divided into six equal-sized groups (n = 12 per group): model control, LY294002 (PI3K/AKT/mTOR inhibitor), 740 Y-P (PI3K/AKT/mTOR agonist), Tuina, Tuina+LY294002, and Tuina+740 Y-P.

#### Model establishment

The KOA rat model was established by intra-articular injection of papain according to the following protocol (30): Activated papain solution was prepared by mixing 4% papain with 0.03 mol/L L-cysteine (2:1 ratio) in sterile saline, followed by activation for 30 min at room temperature. Seventy-eight fasted rats were anesthetized using an intraperitoneal injection of 3% sodium pentobarbital (3 mg/100 g). After right knee hair removal and iodophor disinfection, 0.2 mL of activated solution was injected into the joint cavity through the lateral patellar tendon with the knee flexed to 45°. This procedure was repeated on days 4 and 7, for a total of three injections. After three injections, behavioral changes were assessed using the Lequesne MG rating scale. One rat from each group was randomly selected for HE staining, and the degree of knee cartilage degeneration was evaluated using the Mankin score. A Lequesne MG score of >4 and a Mankin score of ≥6 indicated a successful KOA model construction (31).

#### Intervention methods

**Tuina group:** The rats were immobilized on a fixation plate and the right knee treatment site was exposed. The Tuina intervention was carried out as described previously (20). In summary: (1) Acupressure manipulation: Four acupoints on the right hind limb of rats, Dubi (ST35), Yinlingquan (SP9), Yanglingquan (GB34), and Neixiyan (EX-LE4), were stimulated using a specialized animal acupoint massaging instrument (Patent No. ZL201922406881.8) developed by our research group. Each acupoint received 2 minutes of pressure at a controlled intensity of 3–5 N and a standardized frequency of 60 compressions per minute. (2) Knee flexion and extension: A FingerTPS tactile pressure sensor (PPS Inc., Olathe, KS, USA) was mounted on the operator’s fingers. The rat’s knee joint was initially flexed, followed by the application of downward pressure to the inferior patellar border using the thumb, with concurrent knee extension under a stimulation force of 50 N. This was repeated 10 times. The Tuina intervention was administered daily for 14 consecutive days by a single trained practitioner to ensure treatment consistency and minimize operator-related variability.

LY294002 (PI3K/AKT/mTOR inhibitor): The solution was prepared by dissolving the dry powder of LY294002 successively in dimethyl sulfoxide, polyethylene glycol, polysorbate-80, and normal saline in a ratio of 10:40:5:45, with a concentration of 3.4 mg/mL. The rats received LY294002 solution (20 mg/kg) injected intraperitoneally once a day for 14 consecutive days.

740 Y-P (PI3K/AKT/mTOR agonist): The solution was prepared by dissolving 740 Y-P dry powder successively in pure water and 0.1 mol/L hydrochloric acid. For each mg of powder, 150 μL of water and 30 μL of hydrochloric acid were added and ultrasonic solution-assisted treatment (sonication) was used to bring the concentration of 740 Y-P to 5.5 mg/mL. The rats received 740 Y-P solution (10 mg/kg) injected intraperitoneally once a day for 14 consecutive days.

Tuina+LY294002: The rats received LY294002 solution (20 mg/kg) injected intraperitoneally followed by Tuina therapy once daily for 14 consecutive days.

Tuina+740 Y-P: The rats received 740 Y-P solution (10 mg/kg) injected intraperitoneally followed by Tuina treatment once daily for 14 consecutive days.

Blank group and model group: The blank group and the model control group were administered normal saline (1 mL/kg) once daily for 14 consecutive days.

#### Behavioral assessment

PWT: Joint pain severity was quantitatively assessed using an electronic von Frey apparatus. Rats were individually acclimated in metal mesh cages for ≥30 min to ensure unrestricted paw access and behavioral stabilization. During testing, a calibrated probe was applied perpendicularly to the mid-plantar surface of the right hind paw. The pressure was gradually increased until a nocifensive response (i.e., rapid paw withdrawal, flinching, or jumping) was elicited. The maximum force (g) displayed on the instrument was recorded as the PWT.

KJS: The transverse diameter of the right knee joint was measured using a digital sliding caliper. With the knee flexed at 90°, the horizontal distance between the bilateral prominence points of the femoral condyles was recorded as the KJS index.

PROM: A medical goniometer was used to evaluate the passive mobility of the affected knee joint. The femur was aligned parallel to the fixed arm of the goniometer, ensuring the joint rotational axis coincided with the goniometer pivot. The movable arm tracked tibial movement during passive extension and flexion. The maximal extension and flexion angles were recorded at endpoint positions, and PROM was calculated as the difference between these angles.

All parameters (PWT, KJS, PROM) were measured after the intervention to establish KOA and after 2 weeks of experimental treatment.

### Hematoxylin and eosin staining and Mankin score

After deparaffinization through successive immersions in xylene I and II, tissue sections were rehydrated using a graded ethanol series (absolute ethanol I, II, 95% ethanol, 70% ethanol) followed by distilled water rinses. Sections underwent nuclear staining with modified Lillie-Mayer’s hematoxylin, acid alcohol differentiation (1% HCl in 70% ethanol), and bluing in alkaline tap water. Cytoplasmic counterstaining was performed using eosin Y solution followed by distilled water rinses. After dehydration through an ascending ethanol series (70%, 95%, absolute I, II) and xylene clearing, sections were mounted with neutral balsam. Histopathological evaluation of knee articular cartilage was conducted under light microscopy (200× magnification) using the standard Mankin scoring system, which assesses structural integrity, cellular abnormalities, matrix staining, and tidemark preservation.

### Safranin O/Fast green (SO/FG) staining

The knee tissue of rats was soaked in 4% paraformaldehyde for 24 h. After fixation, the sample was dehydrated with 10% ethylenediaminetetraacetic acid decalcified solution, impregnated with wax, embedded, and sliced into 3-μm sections. After the paraffin sections were dewaxed in water, they were stained with SO/FG, with xylene clearing. Cartilage morphology was systematically analyzed under an optical microscope at 200× magnification, and tissue area quantification was performed using ImageJ software by calculating the percentage of SO-positive cartilage matrix relative to the total articular surface area.

### Transmission electron microscopy

The tissue samples were subjected to standard electron microscopy processing as follows: After thorough cleaning, the specimens were fixed, rinsed, dehydrated, and embedded in resin, followed by oven polymerization. Ultrathin sections (thickness, 60–80 nm) were prepared using an ultramicrotome and mounted on 150-mesh copper grids. Grid-mounted sections were subjected to uranyl acetate staining under light-protected conditions, followed by rinsing and counterstaining with lead citrate in a carbon dioxide–free environment to prevent precipitate formation. Following a final rinse with ultrapure water and complete drying, the samples were examined using TEM. Ultrastructural morphological evaluation was performed at 8,000× magnification. To quantitatively assess the relative autophagy level in chondrocytes, five randomly selected cells were observed under a transmission electron microscope at 3,000× magnification and the number of autophagosomes in each cell were counted. The mean number of autophagosomes in the five cells was calculated and used as an indicator of the relative autophagy level for each sample.

### Reverse-transcription quantitative PCR

The mRNA expression levels of PI3K, AKT, mTOR, ULK1, Beclin-1, LC3II/I, ATG5, ATG7 and P62 in cartilage tissues were detected using reverse-transcription quantitative PCR (RT-qPCR). The methods and steps were as follows: The cartilage tissue was ground in liquid nitrogen, lysis buffer was added, homogenized with a homogenizer, chloroform was added, centrifuged, mixed with anhydrous ethanol, transferred to an adsorption column, centrifuged, and protein-removing solution and bleaching solution were added successively, centrifuged, the waste liquid was discarded, 50μL of RNase-Free dd H_2_O was added, and centrifuged. After detecting the purity and concentration of mRNA with an ultramicro nucleic acid analyzer, reverse-transcription reactions were carried out: 2μL of RNA was taken for PCR amplification, a 20-μL reverse-transcription reaction system was prepared, and the relative expression levels of each gene were calculated using the 2-ΔΔCt method. The primer sequences are listed in **Table 1**.

**Table 1:** RT-qPCR primer sequences for target genes.

Gene	Forward primer (5′–3′)	Reverse primer (5′–3′)
PI3K	CTGCTGCAAAACCCCATCAC	AGCGGTGGTCTATCAACAGC
AKT	ATGGACTCAAACGGCAGGAG	AGCACCTGAGTTGTCACTGG
mTOR	CACCAAGGCCTAATGGGGTT	CAACAACGGCTTTCCACCAG
P62	ATGAGAGACAAAGCCAAGGAGG	CATGGGGGTCCAAAGACTTCA
ATG5	GGGACTGCAGAATGATTTGACC	GAAAGGCCGTTCAGTTGTGG
ATG7	GGCTAGAACACTGATGGGCT	GCCTCACGGGATTGGAGTAG
ULK1	TACACACCCTCTCCCCAAGT	GTGCTCAGGCACAGAGGAG
Beclin-1	AGGAGAGAGCCAGGAGGAAG	GACACCATCCTGGCGAGTTT
LC3-II	GAAGACCTTCAAACAGCGCC	ATCACTGGGATCTTGGTGGG
LC3-I	AAGACCGGTCAGAAGCCATC	AGCAAGTGTGGACAGAGACG
GAPDH	TGATGCCCCCATGTTTGTGA	TTCTGAGTGGCAGTGATGGC

### Western blot

The expression levels of PI3K, AKT, mTOR, and downstream autophagy-related proteins (ULK1, Beclin-1, LC3II/I, ATG5, ATG7, and P62) in rat articular cartilage tissues were measured using WB. The experimental procedure was conducted as follows: Proteins were first extracted from cartilage tissues and quantified using the BCA assay. Equal amounts of protein samples (30 μg per lane) were separated on 10% Sodium dodecyl-sulfate polyacrylamide gel electrophoresis (SDS-PAGE) gels through electrophoresis at 80 V for 2 h, followed by semi-dry transfer onto PVDF membranes. Membranes were subsequently blocked with 5% non-fat milk in TBST for 1 h at room temperature before incubation with primary antibodies (diluted 1:1000 in blocking buffer) overnight at 4 °C. Following three 10-minute TBST washes, membranes were incubated with HRP-conjugated secondary antibodies (1:5000 dilution) for 1 h at room temperature. After additional washing steps, protein bands were visualized using enhanced chemiluminescence substrate and quantified through densitometric analysis with ImageJ software. GAPDH served as the loading control for normalization of target protein expression levels.

### Immunohistochemistry

Immunohistochemical analysis was performed to assess the levels of PI3K, AKT, mTOR, ULK1, Beclin-1, LC3II/I, ATG5, ATG7, and P62 protein expression in rat articular cartilage tissues using a standardized protocol. Paraffin-embedded sections were first subjected to deparaffinization and rehydration through sequential immersion in xylene I and II, followed by absolute ethanol I and II, and a graded ethanol series. Antigen retrieval was achieved using protease-based antigen repair solution under optimized incubation conditions. Subsequent procedures included peroxidase activity blocking with 3% H_2_O_2_, glycine buffer treatment, and non-specific binding site blocking with 5% BSA. Sections were then incubated overnight at 4 °C with specific primary antibodies (1:200 dilution), followed by appropriate HRP-conjugated secondary antibodies (1:500 dilution) at room temperature for 1 h. Chromogenic development was performed using DAB substrate, with hematoxylin counterstaining for nuclear visualization. After dehydration through an ethanol gradient and xylene clearing, sections were mounted with neutral balsam. Image acquisition was conducted using the Motic3000 digital photomicrograph system at 400× magnification, with quantitative analysis of positively stained cells performed using Image-Pro Plus 6.0 software.

### Statistical analysis

Statistical analyses were performed using SPSS (version 26.0; IBM Corp., Armonk, NY, USA) and GraphPad Prism 8.0 (GraphPad Software, San Diego, CA, USA), with the latter used for graphical representation. All datasets were subjected to comprehensive assessments of normality (with significance defined as *P* > 0.05) and homogeneity of variance using Levene’s test. All data were expressed as mean ± standard deviation (SD). The analytical approach was applied as follows:

1. One-way analysis of variance (ANOVA) and Levene’s test to assess the homogeneity of variance: If the data were normally distributed, homogeneity of variance was assessed using Levene’s test. If variance homogeneity was confirmed (*P* > 0.05) and the ANOVA indicated significant intergroup differences (*P* < 0.05), *post-hoc* pairwise comparisons were conducted using the Least Significant Difference (LSD) test.
2. Welch’s F-test for heterogeneous variance: If the data were normally distributed but exhibited unequal variance (as indicated by Levene’s test, *P* ≤ 0.05), Welch’s F-test was applied. If significant differences were detected (*P* < 0.05), pairwise comparisons were performed using the LSD test.
3. Kruskal–Wallis Test for Non-Normal Data: For datasets that deviated from normality, the non-parametric Kruskal–Wallis test was used. When overall significance was observed (*P* < 0.05), subsequent pairwise comparisons were performed using Wilcoxon rank-sum tests.

For all pairwise comparisons, the LSD method was applied, and the Bonferroni correction was used to adjust for multiple comparisons. Two-sided *P* values less than 0.05 were considered statistically significant.

### Machine learning

#### Data sets and preprocessing

We selected 15 intraexperimental indicators as input features for the ML model. These variables included behavioral assessments (PWT, KJS, and PROM), cartilage degeneration measures (Mankin score and cartilage area ratio [CAR]), autophagy-related indicators (RAL, P62, ULK1, ATG7, ATG5, Beclin-1, and LC3II/LC3I levels), and PI3K/AKT/mTOR-related indicators (PI3K, AKT, and mTOR levels).

To assess multicollinearity, we calculated the variance inflation factor (VIF) for all candidate features. Features with a VIF >10 were excluded. The final 15 features had VIF values ranging from 1.5 to 4.7, confirming minimal inter-feature redundancy (32). Text labels in the *in vivo* experimental data were converted into digital labels using one-hot encoding, and the dataset was normalized (z-score normalization) to ensure uniform feature scaling. The selected features were then analyzed to simplify the number of features and remove features with poor predictive value, thereby improving the stability of model training.

#### Model construction

An SVM model was constructed in Python 3.12.3 using the using the sklearn. svm module in Scikit-learn. This model was based on the principles of the maximum margin classification and kernel methods (33, 34). The SVM algorithm identified an optimal hyperplane in the feature space to separate the data points of different classes. This hyperplane was determined by support vectors, which are the data points closest to the decision boundary, with the margin representing the distance between the hyperplane and the nearest instances. The objective of the model was to maximize the margin to achieve optimal class separation.

The algorithm used kernel functions (including linear, polynomial, and radial basis function [RBF] kernels) to transform data from the original feature space into higher-dimensional representations, facilitating the identification of separable hyperplanes (35). The selection of the kernel type and associated parameters (such as the polynomial degree or RBF gamma value) significantly influences model performance (36, 37). To ensure reproducibility, hyperparameter optimization was conducted using a grid search approach with 5-fold cross-validation. The evaluated parameter ranges were as follows: C = [1, 5, 10, and 20], gamma = [0.01, 0.1, 1, and 10], and kernel = [linear, RBF, and polynomial]. The optimal configuration was kernel type = RBF, regularization parameter (C) = 10, and kernel coefficient (gamma) = 0.1.

The model was optimized by using quadratic programming to simultaneously maximize the margin and minimize the number of classification errors. After determining the optimal hyperparameters, the final SVM model was trained using 70% of the normalized dataset (training set) and tested on 30% (test set) to avoid overfitting. Its performance on the test set was used to evaluate the correlation between different evaluation measures and predict the overall and categorical efficacy of treatment measures (38).

#### Model evaluation

Initially, the performance of the SVM model was evaluated using three key metrics: accuracy, F1 score, and Cohen’s kappa coefficient. Accuracy quantified the proportion of correctly classified samples relative to the total test set and serves as a fundamental measure in classification tasks (39). The F1 score (harmonic mean of precision and recall) and kappa coefficient (inter-rater agreement measurement) provided complementary assessments of classification consistency with the ground truth. A comprehensive performance analysis was conducted using a confusion matrix, in which rows represented actual classes and columns indicated predicted classifications, with matrix cells containing sample counts for each actual–predicted class combination (40). To enhance interpretability, we implemented matrix normalization and generated visual heatmap representations.

After validating the effectiveness of the SVM model, we leveraged its optimal hyperplane separation principle to conduct goodness-of-fit tests on 15 standardized indicator sets and their combinations. The R-squared (R^2^) metric was used to measure the correlation between the individual indicators and their combined effects (41, 42).

We used the data of the blank control and model groups as the training and testing sets, respectively. Subsequent predictions were made for the remaining five experimental interventions to assess their therapeutic efficacy. Two levels of efficacy evaluation were conducted for each indicator: overall efficacy analysis and classified therapeutic effects analysis of the index type (behavioral assessments, cartilage degeneration measures, and autophagy- and PI3K/AKT/mTOR-related indicators).

## Results

### Tuina ameliorates mechanical pain, joint swelling, and functional activity in KOA rats

**Figure 2:**
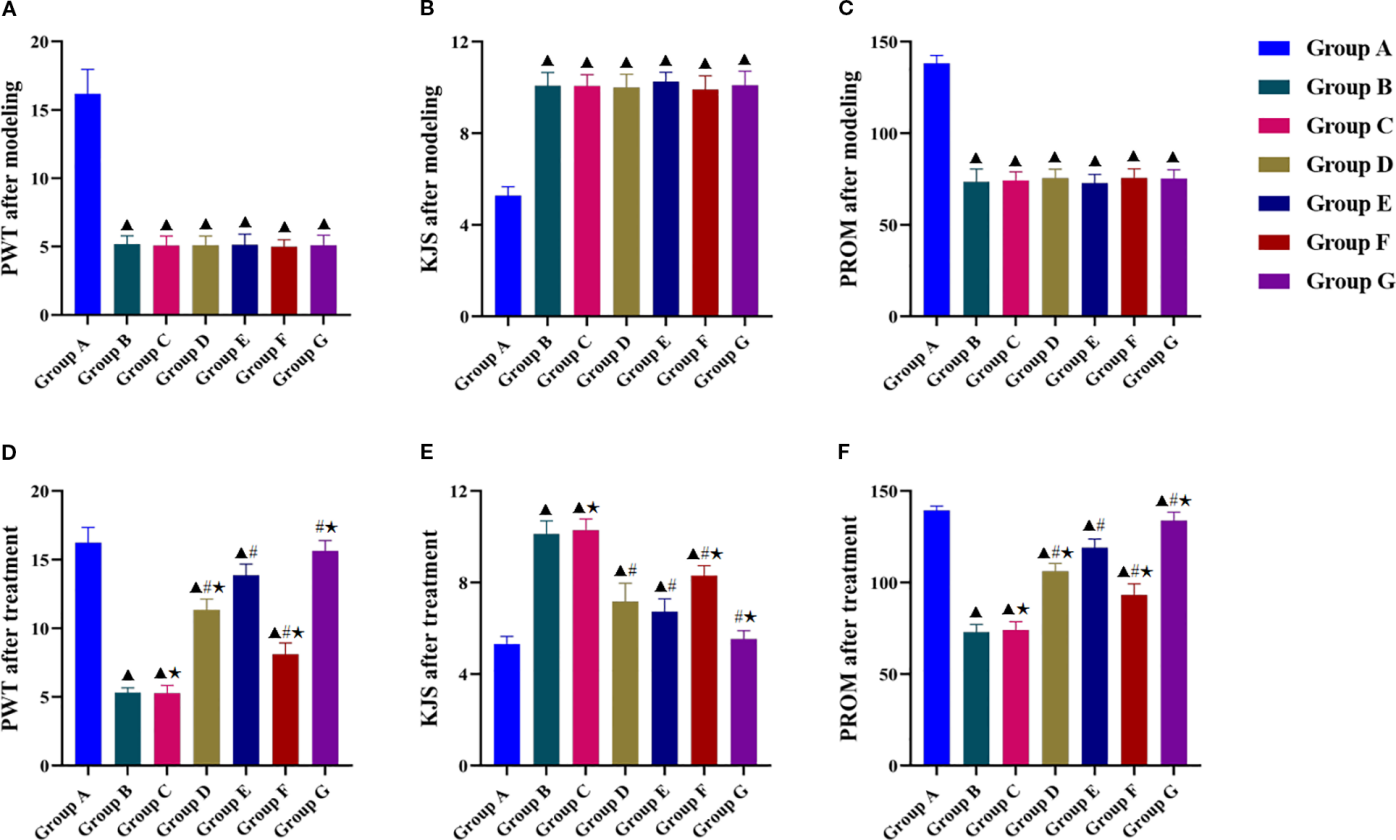
Effect of Tuina on behavioral indicators of the knee joint in KOA rats. (**A–C**, PWT, KJS and PROM after modeling of KOA rats in each group; **D–F**, PWT, KJS and PROM after treatment. Data are expressed as mean ± SD. Group A, blank group; Group B, model group; Group C, LY294002 group; Group D, 740 Y-P group; Group E, Tuina group; Group F, Tuina+LY294002 group; Group G, Tuina+740 Y-P group. ^▴^ Compared with group A, *P* < 0.05; ^#^ Compared with group B, *P* < 0.05; ★ Compared with group E, *P* < 0.05.

Post-modeling assessments revealed significant differences in PWT, KJS, and PROM between the experimental groups and the blank control group (**Figures 2A–C**), confirming successful KOA induction. Post-intervention analysis demonstrated significantly greater improvements in PWT, KJS, and PROM in all treatment groups than in the model group (**Figures 2D–F**), but the improvement was significantly less in the LY294002 group than in the other four intervention groups. Tuina+740 Y-P combination therapy resulted in significantly better PWT, KJS, and PROM outcomes than those of Tuina monotherapy, whereas Tuina+LY294002 combination therapy was significantly less effective than that of Tuina monotherapy. Tuina and 740 Y-P monotherapy had similar anti-edema effects on KJS, whereas Tuina monotherapy showed significantly better performance than that of 740 Y-P monotherapy in restoring PROM and improving the PWT.

### Tuina mitigates cartilage lesions and reduces KOA progression in rats

**Figure 3:**
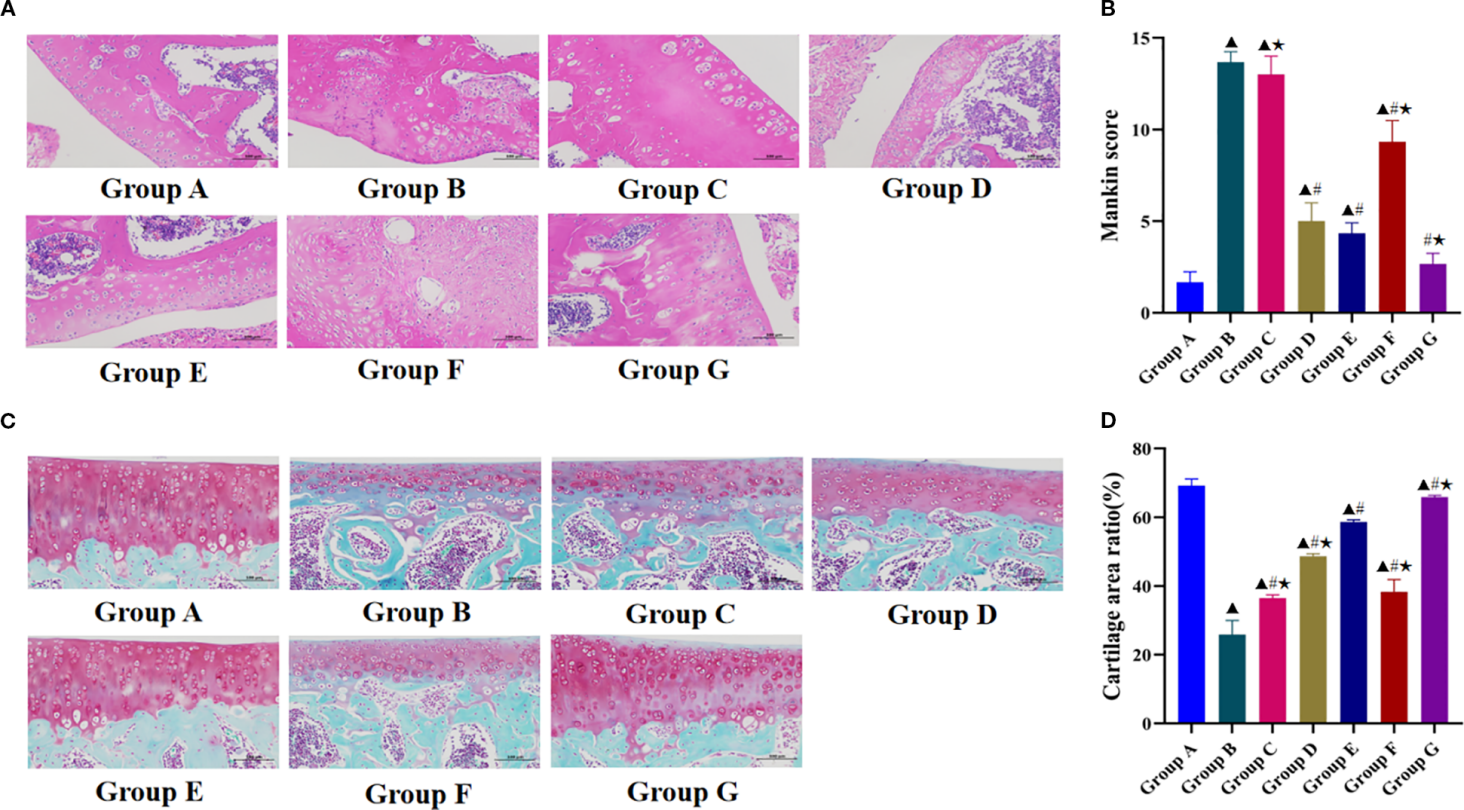
Effect of Tuina on the degeneration of knee cartilage in KOA rats. **(A)**, Observation results of HE stained knee cartilage of rats in each group under light microscope; **(B)**, Mankin score of chondrocytes in each group; **(C)**, Observation results of SO/FG stained knee cartilage of rats in each group under light microscope; **(D)**, Cartilage area ratio in each group. Data are expressed as mean ± SD. Group A, blank group; Group B, model group; Group C, LY294002 group; Group D, 740 Y-P group; Group E, Tuina group; Group F, Tuina+LY294002 group; Group G, Tuina+740 Y-P group. ^▴^ Compared with group A, *P* < 0.05; ^#^ Compared with group B, *P* < 0.05; ★ Compared with group E, *P* < 0.05.

HE staining revealed distinct histological differences among the experimental groups (**Figure 3A**). In the blank group, articular cartilage exhibited a smooth surface with abundant chondrocytes and homogeneous matrix distribution, accompanied by well-defined subchondral bone and synovial membrane structures. In contrast, the model group showed marked pathological alterations characterized by surface irregularities, focal pannus formation, severe cartilage erosion with structural disruption, necrotic chondrocyte dissolution presenting as amorphous eosinophilic material, and indistinct tidemark structure. The degree of cartilage repair and degeneration reversal relative to that of the model group was greatest in the Tuina+740 Y-P group, followed by the Tuina monotherapy and 740 Y-P monotherapy groups. These three groups showed marked restoration of cartilage structure with increased chondrocyte populations and improved matrix organization. The Tuina+LY294002 group showed the least improvement, exhibiting only marginal increases in chondrocyte numbers and partial matrix realignment, and substantial residual cartilage destruction. The LY294002 group did not show any therapeutic benefits, maintaining pathological features similar to those of the model group.

The Mankin scores were significantly higher in the model group than in the blank group (**Figure 3B**), confirming the successful induction of KOA. All therapeutic intervention groups except the LY294002 group had significantly lower Mankin scores than that of the model group. Furthermore, the Tuina+740 Y-P combination group had significantly better therapeutic outcomes and lower Mankin scores than those of the Tuina monotherapy group, whereas the Tuina+LY294002 group had significantly higher pathological scores than those of the Tuina monotherapy group.

The SO/FG staining results revealed distinct morphological differences among experimental groups (**Figure 3C**). The blank control group maintained intact articular cartilage architecture featuring a preserved superficial layer, continuous surface regularity, physiological cartilage thickness, and normal chondrocyte distribution. In marked contrast, the model group exhibited marked pathological degeneration characterized by extensive superficial cartilage erosion, substantial depletion of extracellular matrix components, pronounced chondrocyte loss, and reduced cartilage thickness. All intervention groups demonstrated varying degrees of enhanced cartilage repair compared with that of the model group. The Tuina+740 Y-P combination group had the best clinical outcomes, with near-complete cartilage structural restoration with chondrocyte repopulation and cartilage thickness approximating normal control levels. The Tuina monotherapy and 740 Y-P monotherapy groups had intermediate clinical outcomes, whereas the LY294002 group had the worst outcomes, showing only a slight increase in cartilage thickness.

All intervention groups had significantly higher CAR values than that of the model group (**Figure 3D**). Furthermore, the Tuina+740 Y-P group had a significantly greater CAR value than that of the Tuina monotherapy group, whereas the 740 Y-P monotherapy, Tuina+LY294002, and LY294002 monotherapy groups had significantly lower CAR values than that of the Tuina monotherapy group.

These divergent therapeutic outcomes demonstrate that combined application of 740 Y-P and Tuina therapy had a synergistic effect on cartilage repair, whereas co-administration of the PI3K/AKT/mTOR signaling inhibitor LY294002 significantly attenuated the therapeutic effects of Tuina therapy on chondral regeneration. These results confirm that the PI3K/AKT/mTOR signaling pathway is involved in mediating the effects of Tuina therapy on the cartilage repair process.

### Tuina inhibits excessive chondrocyte autophagy in KOA rats

**Figure 4:**
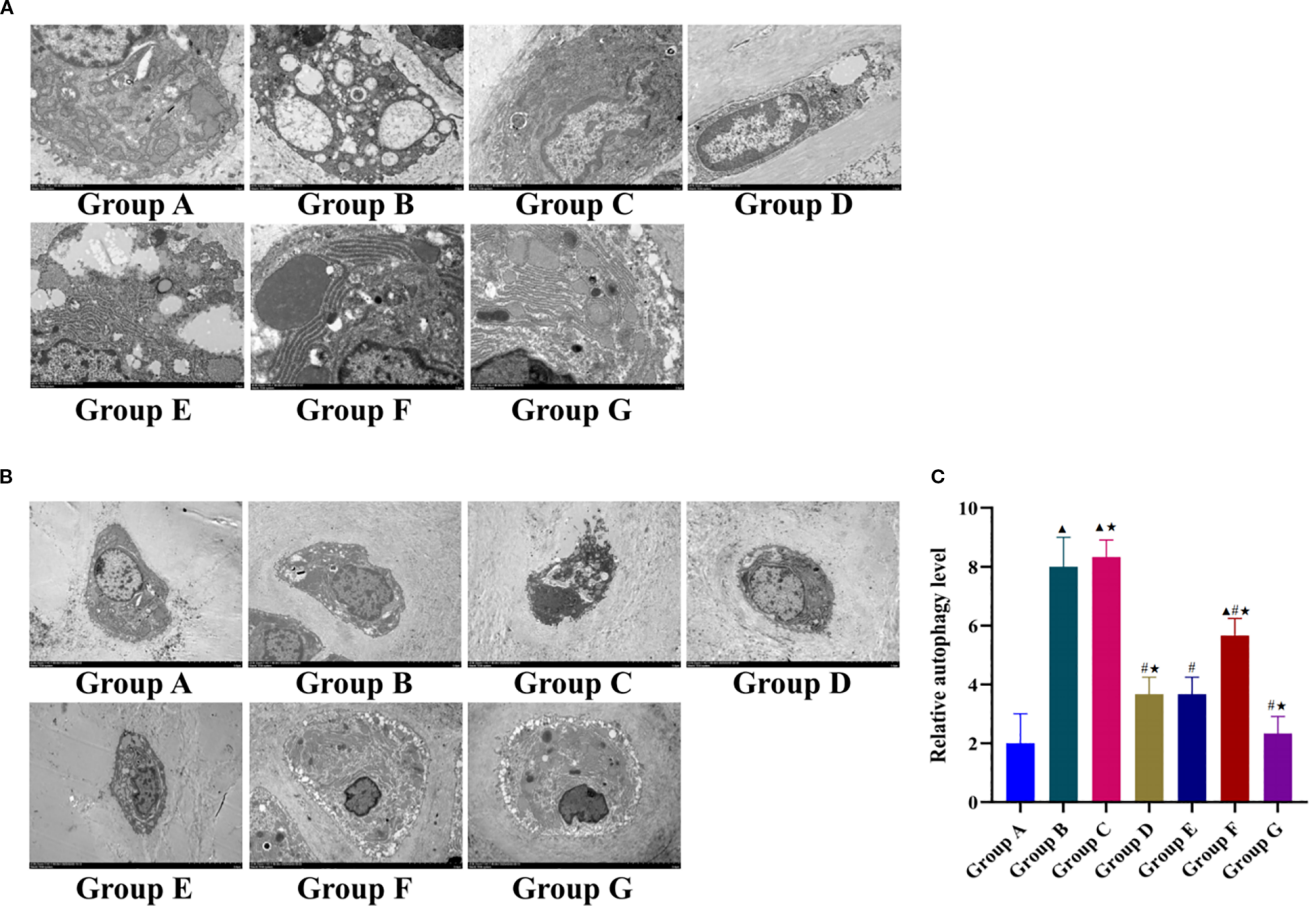
Effect of Tuina on autophagy of knee chondrocytes in KOA rats. **(A)**, Observation results of knee cartilage of rats in each group under 8000x TEM; **(B)**, 3000x TEM; **(C)**, Relative autophagy level in each group. Data are expressed as mean ± SD. Group A, blank group; Group B, model group; Group C, LY294002 group; Group D, 740 Y-P group; Group E, Tuina group; Group F, Tuina+LY294002 group; Group G, Tuina+740 Y-P group. ^▴^ Compared with group A, *P* < 0.05; ^#^ Compared with group B, *P* < 0.05; ★ Compared with group E, *P* < 0.05.

We used TEM to observe the ultramorphology of cartilage and quantify the relative autophagy level. The ultramorphological changes in articular chondrocytes in each group are shown at 8000× magnification in **Figure 4A**. In the blank control group, chondrocytes displayed characteristic physiological architecture with intact plasma membranes, dense perinuclear extracellular matrixes, and well-preserved organelles. Nuclei maintained regular contours, mitochondria exhibited intact membranes with distinct cristae, and rough endoplasmic reticulum (RER) demonstrated abundant ribosome attachment. Autophagosomes and lysosomes were present at baseline physiological levels. The model group exhibited marked degenerative changes including cellular shrinkage, matrix dissolution with delicate fibrillar network disintegration, and organelle pathology, including nuclear membrane invagination, mitochondrial depletion with cristae fragmentation, and dilated RER cisternae, and a significant increase in the number of autophagic vacuoles (autophagosomes and autolysosomes) compared with that in the controls. The LY294002 group showed no improvement, maintaining pathology comparable to that of the model group.

The Tuina+740 Y-P intervention group showed substantial ultrastructural recovery: Plasma membranes regained integrity with short cytoplasmic projections, nuclei exhibited regular morphology with chromatin condensation, and mitochondrial architecture showed restored cristae organization with reduced matrix dilution. RER structures normalized with ribosome reattachment, and autophagic vacuoles decreased to near-physiological levels. The Tuina monotherapy and 740 Y-P alone groups had moderate improvements in their cellular architecture and autophagy regulation, which was less marked than those in the combination therapy group. The Tuina+LY294002 group showed partial mitigation of pathology, including reduced RER dilation and moderate autophagosome decrease, but persistent abnormalities such as cytoplasmic contraction, organelle swelling, mitochondrial membrane dissolution, and nuclear membrane blurring.

TEM at 3000× magnification revealed significant intergroup differences in the number of autophagosomes (**Figures 4B, C**). Compared with the blank control group, the model group had a significantly higher number of autophagosomes, whereas all intervention groups except the LY294002 group had significantly fewer autophagosomes than that of the model group. Notably, the Tuina+740 Y-P combination had significantly fewer autophagosomes than that of the Tuina monotherapy group. Although the 740 Y-P monotherapy group had comparable autophagic regulation to that of the Tuina monotherapy group, the Tuina+LY294002 group displayed incomplete autophagy suppression, and a significantly higher number of autophagosomes than that in the Tuina monotherapy group.

### Tuina inhibits chondrocyte autophagy in KOA rats by promoting the PI3K/AKT/mTOR signaling pathway

**Figure 5:**
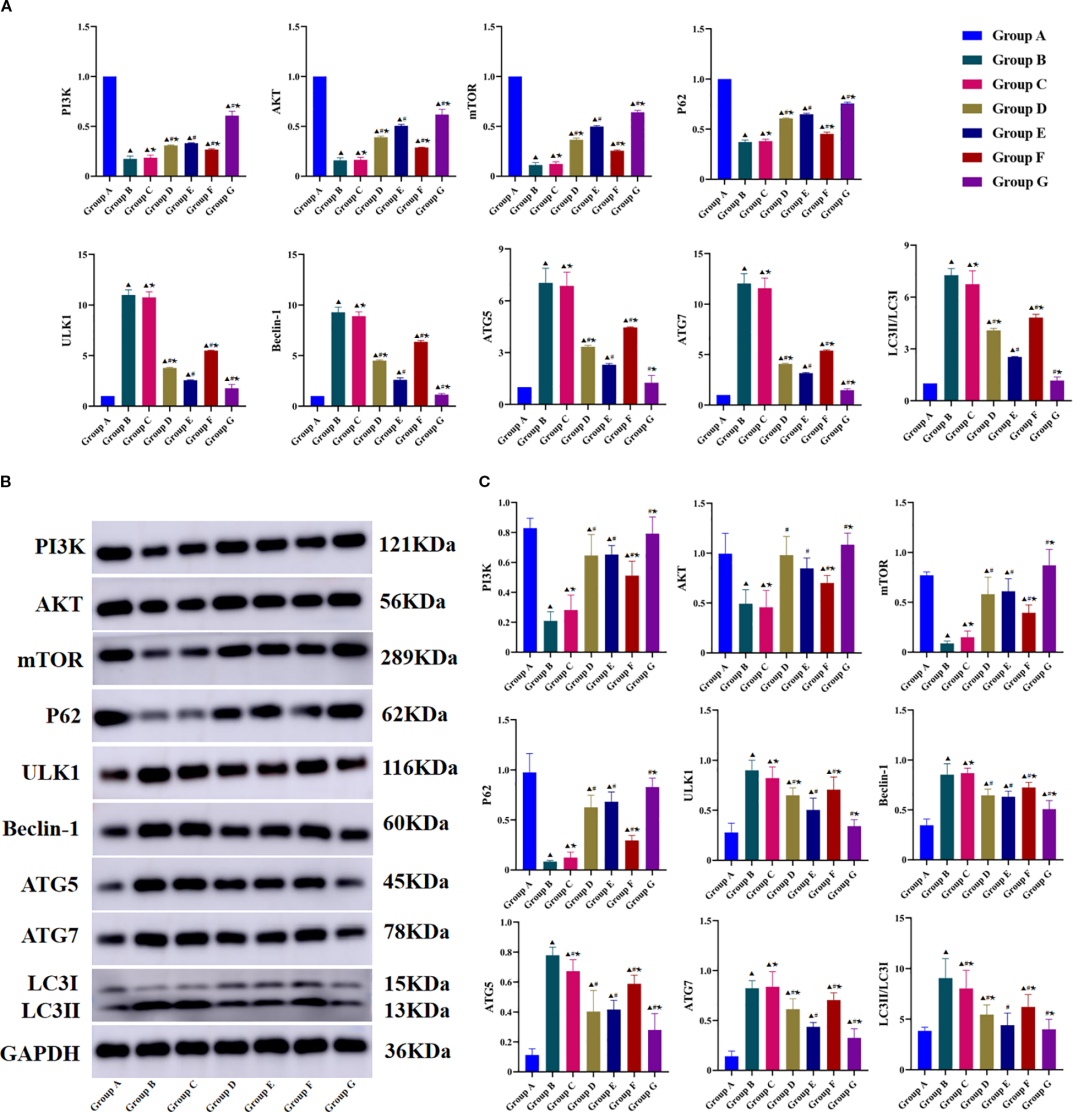
Effects of Tuina on the expressions of PI3K/AKT/mTOR signaling pathway-Related in Knee Cartilage Tissues of KOA Rats. **(A)**, Result of RT-qPCR in each group; **(B)**, Result of Western blot band; **C**, Result of western blot statistical analysis. Data are expressed as mean ± SD. Group A, blank group; Group B, model group; Group C, LY294002 group; Group D, 740 Y-P group; Group E, Tuina group; Group F, Tuina+LY294002 group; Group G, Tuina+740 Y-P group. ^▴^ Compared with group A, *P* < 0.05; ^#^ Compared with group B, *P* < 0.05; ★ Compared with group E, *P* < 0.05.

The PI3K/AKT/mTOR signaling pathway is a central regulator of autophagy initiation. We hypothesized that the autophagy-inhibitory effect of Tuina intervention in chondrocytes of KOA rats may be mediated through PI3K/AKT/mTOR pathway modulation. PCR and WB analysis demonstrated significant downregulation in both mRNA and protein expression levels of PI3K, AKT, and mTOR in the model group compared with those in the blank control group (**Figures 5A-C**). Tuina therapy resulted in significantly increased PI3K, AKT, and mTOR expression compared with that of the untreated model group. Co-treatment with the PI3K activator 740 Y-P enhanced the effect of Tuina, resulting in significantly higher PI3K, AKT, and mTOR expression than that in the Tuina monotherapy group. Conversely, administration of the PI3K inhibitor LY294002 attenuated Tuina-mediated upregulation, resulting in significantly lower PI3K, AKT, and mTOR expression than that in the Tuina monotherapy group.

Downstream autophagy-related biomarkers were analyzed using RTqPCR and WB (**Figures 5A-C**). Compared with the model group, the Tuina group had significantly increased P62 mRNA and protein expression and significantly decreased ULK1, Beclin-1, LC3-II/LC3-I, ATG5, and ATG7 expression. The Tuina+740 Y-P combination group showed significantly greater reductions in Beclin-1, ULK1, LC3-II/LC3-I, ATG5, and ATG7 expression, and significantly greater P62 expression compared with that of the Tuina monotherapy group. In contrast, the Tuina+LY294002 combination group showed significantly less reduction in Beclin-1, ULK1, LC3-II/LC3-I, ATG5, and ATG7 expression, and significantly less P62 expression compared with that of the Tuina monotherapy group. These results confirm the functional involvement of PI3K/AKT/mTOR signaling in mediating the therapeutic effects of Tuina on regulating chondrocyte autophagy.

**Figure 6:**
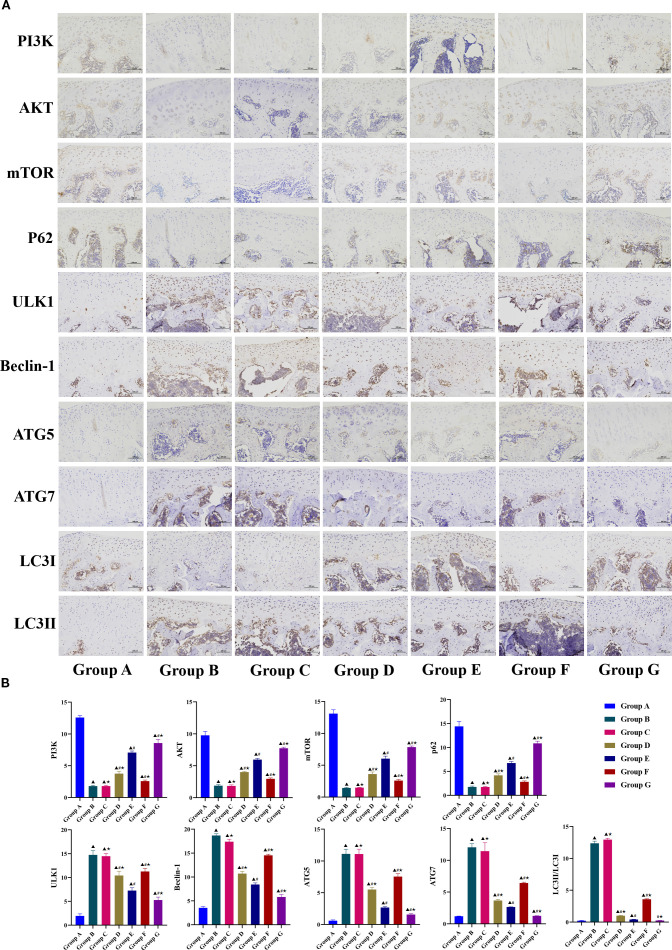
Immunohistochemical analysis results of PI3K/AKT/mTOR signaling pathway-related indicators. **A**, Observation results of immunohistochemistry in each group; **B**, Positive expression rate. Data are expressed as mean ± SD. Group A, blank group; Group B, model group; Group C, LY294002 group; Group D, 740 Y-P group; Group E, Tuina group; Group F, Tuina+LY294002 group; Group G, Tuina+740 Y-P group. ^▴^ Compared with group A, *P* < 0.05; ^#^ Compared with group B, *P* < 0.05; ★ Compared with group E, *P* < 0.05.

Immunohistochemical analysis further corroborated the findings from WB and PCR assays (**Figures 6A, B**). Compared with the model group, the LY294002 group had a significantly lower Beclin-1 positive expression rate, but the other markers did not differ significantly between the two groups. Other treatment groups had significantly higher expression of PI3K, AKT, mTOR, and P62, and significantly lower expression of ULK1, Beclin-1, LC3-II/LC3-I, ATG5, and ATG7. Compared with the Tuina monotherapy group, the Tuina+LY294002 combination group showed significantly lower expression of PI3K, AKT, mTOR, and P62, and significantly lower ULK1, Beclin-1, LC3-II/LC3-I, ATG5, and ATG7 levels. Conversely, the Tuina+740 Y-P group showed significantly higher expression of PI3K, AKT, mTOR, and P62, and ULK1, Beclin-1, LC3-II/LC3-I, ATG5, and ATG7 levels than those in the Tuina monotherapy group. Collectively, these data suggest that Tuina therapy may inhibit chondrocyte autophagy in KOA rats by activating the PI3K/AKT/mTOR signaling pathway.

### ML confirms that Tuina mitigates KOA by activating PI3K/AKT/mTOR signaling pathway-mediated autophagy

**Figure 7:**
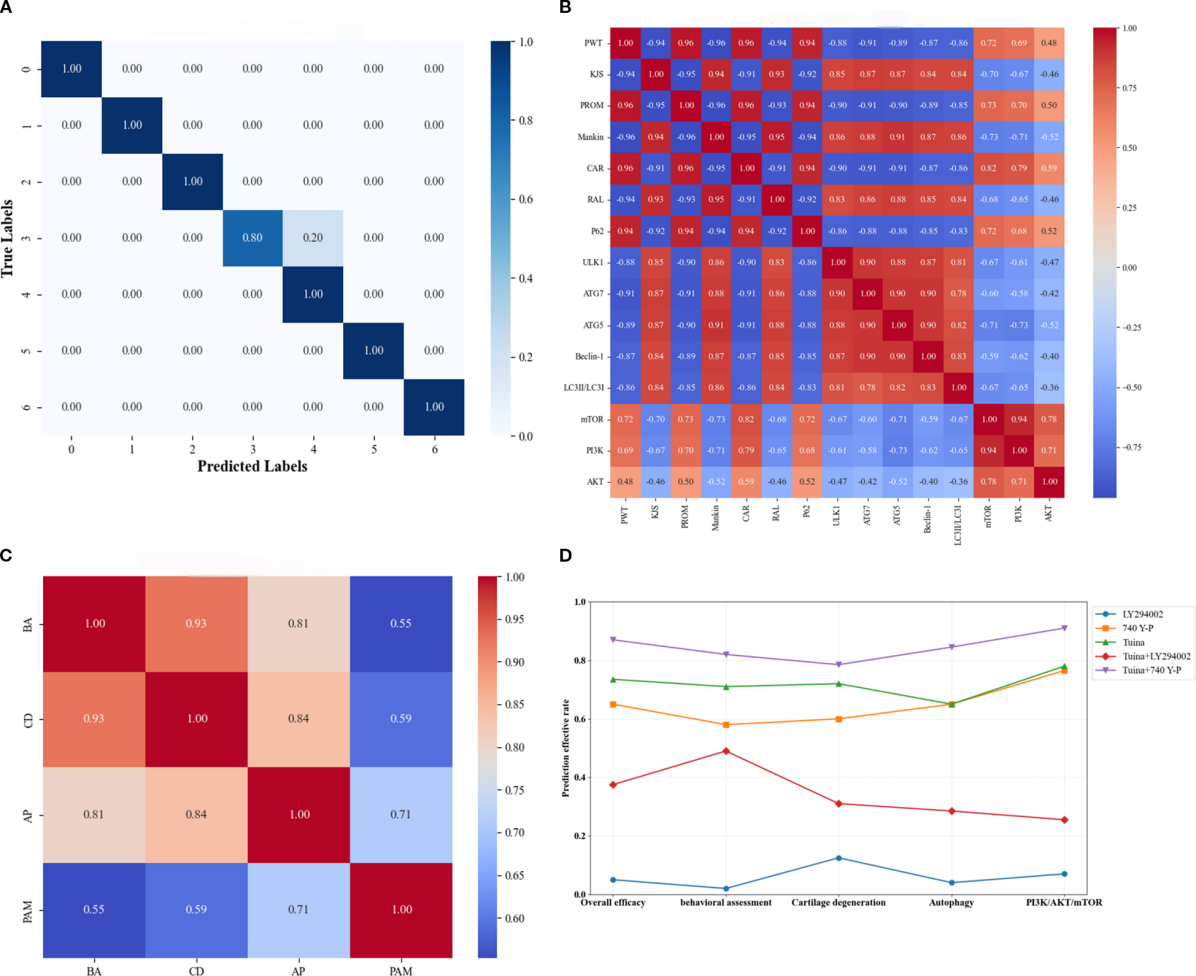
Results of the support vector machine algorithm under the machine learning framework. **(A)**, Visual confusion matrix of model validity; **(B)**, Individual correlation analysis; **(C)**, Categorical correlation analysis; **(D)**, Comprehensive and classified therapeutic efficacy analysis. BA, Behavioral assessment; CD, Cartilage degeneration; AP, Autophagy; PAM, PI3K/AKT/mTOR signaling pathway.

We used an SVM model to assess synergistic interactions among biological indicators and predict comprehensive therapeutic outcomes of intervention strategies. The SVM model had 97.62% classification accuracy, and the results of the univariate and multivariate analyses were consistent. This performance was further supported by F1 scores and Cohen’s kappa coefficient. Specifically, the seven-class classification achieved F1 scores of 1.00, 1.00, 1.00, 0.89, 0.91, 1.00, and 1.00 for respective categories, respectively, with an overall kappa coefficient of 0.967 indicating near-perfect inter-rater agreement. Model efficacy was additionally confirmed through confusion matrix analysis (**Figure 7A**).

In the individual correlation analysis PROM and PWT showed the strongest positive correlations with CAR (0.96) and the strongest negative correlations with Mankin scores (−0.96) (**Figure 7B**). The PI3K/AKT/mTOR signaling components exhibited tight intra-pathway connectivity, particularly between PI3K and mTOR (0.94). Although downstream biomarkers P62 and ATG5 displayed moderate associations with this pathway, other biomarkers showed limited connectivity. Notably, autophagy-related biomarkers correlated strongly with both CAR and RAL, although P62 demonstrated the opposite trend to that of the other autophagy markers. Categorical correlation analysis revealed that behavioral assessments showed the strongest association with cartilage degeneration (0.93) and the weakest association with PI3K/AKT/mTOR pathway activity (0.55) (**Figure 7C**). Autophagy displayed dual strong correlations with both cartilage degeneration (0.84) and behavioral assessments (0.81). Furthermore, a significant cross-talk was observed between PI3K/AKT/mTOR signaling and autophagy regulation (0.71).

Compared with the LY294002 group, all other intervention groups had better overall therapeutic outcomes (**Figure 7D**). Compared with the Tuina monotherapy group, the Tuina+740 Y-P combination group had significantly better comprehensive and categorized therapeutic outcomes, whereas the Tuina+LY294002 group had significantly poorer comprehensive and categorized therapeutic outcomes than those of the Tuina monotherapy group. Notably, although the Tuina monotherapy group outperformed the 740 Y-P group in improving comprehensive therapeutic outcomes, behavioral assessments, and cartilage degeneration, both groups demonstrated comparable efficacy in modulating cellular autophagy and the PI3K/AKT/mTOR signaling pathway.

These findings suggest that Tuina manipulation may exert its therapeutic effects on KOA through the regulation of PI3K/AKT/mTOR-mediated autophagy mechanisms, thereby effectively delaying cartilage degeneration progression.

## Discussion

KOA is characterized by progressive cartilage degeneration and primarily driven by dysregulation in chondrocyte autophagy (43, 44). The pathogenesis of KOA involves autophagy abnormalities that disrupt chondrocyte homeostasis, impair secretion of cartilage-specific extracellular matrix components critical for joint function, and ultimately accelerate cartilage destruction. The PI3K/AKT/mTOR pathway has been shown to regulate chondrocyte autophagy, and this has emerged as a key focus of KOA research (45). Pathway inhibition activates downstream autophagy-related molecules including ULK1, ATG5, and LC3, leading to massive autophagosome formation that subsequently combines with lysosomes to execute autophagy (46, 47). Most previous mechanistic investigations of Tuina therapy for KOA have predominantly focused on inflammatory responses and biomechanical factors, with limited attention to chondrocyte autophagy. Our preliminary studies have suggested potential associations between Tuina intervention and modulation of the PI3K/AKT/mTOR signaling axis and autophagy processes, though direct targeting of this pathway remains unconfirmed (20). Moreover, our prior experimental observations were confined to isolated outcome measures, overlooking the dynamic regulatory networks and interdependent relationships among various biological indicators. To address these research gaps, we propose a comprehensive investigation combining PI3K/AKT/mTOR pathway modulators (both inhibitors and activators) with *in vivo* rat experiments and ML approaches. This integrated strategy aims to systematically elucidate the autophagy-mediated mechanisms underlying Tuina therapy in KOA management.

The rat KOA model using L-cysteine-activated papain injection simulated early-stage human KOA pathology through papain-induced degradation of proteoglycans in the cartilage matrix (48, 49). Our study evaluated a therapeutic Tuina massage protocol that combined acupoint compression and passive knee mobilization, using a quality-controlled device to standardize the manipulation techniques. Based on extensive preliminary and formal experiments, we found that applying acupressure at a force of 3–5 N along with a passive knee extension force of 50 N yielded the optimal intervention outcomes in KOA rats. The acupoint pressure technique focused on two complementary pairs from the yin-yang meridian pairing system: Dubi (ST35) with Neixiyan (EX-LE4), and Yinlingquan (SP9) with Yanglingquan (GB34). These antagonistic meridian pairings embody the Traditional Chinese Medicine (TCM) principle of yin-yang harmonization, demonstrating synergistic therapeutic effects through homeostatic regulation in KOA management (50, 51). Precise pressure application at these acupoints facilitates multiple therapeutic outcomes: promoting qi-blood circulation, releasing soft tissue adhesions, enhancing microcirculation, and improving knee joint stability and mobility (52). Concurrently, controlled passive knee mobilization induces biomechanical adjustments that effectively expand joint space dimensions and redistribute intra-articular stress concentration (53). This dual-modality approach achieves classical TCM objectives by restoring proper bone alignment and soft tissue flexibility through integrated therapeutic mechanisms.

740 Y-P and LY294002 represent two pivotal compounds in PI3K/AKT/mTOR signaling pathway research with diametrically opposed mechanisms of action. 740 Y-P functions as a cell-permeable PI3K/AKT/mTOR pathway activator, exerting its biological effects by specifically binding to the N- and C-terminal SH2 domains of the p85 regulatory subunit (54). This interaction mimics phosphotyrosine-mediated recruitment mechanisms, thereby potentiating PI3K activation and subsequent downstream signaling. In contrast, LY294002 serves as a classic PI3K inhibitor that effectively suppresses downstream AKT phosphorylation through competitive inhibition of PI3K’s ATP-binding catalytic domain (55). In this study, we administered 740 Y-P or LY294002 in conjunction with Tuina therapy to investigate alterations in the PI3K/AKT/mTOR signaling pathway, cellular autophagy, cartilage degeneration, and behavioral indices. This experimental design aimed to determine whether the mechanism by which Tuina therapy modulates chondrocyte autophagy to delay cartilage degeneration is directly mediated through PI3K/AKT/mTOR pathway targeting.

Both mRNA and protein expression levels of PI3K, AKT, and mTOR were reduced in cartilage tissue in the model and LY294002-treated groups, indicating suppression of the PI3K/AKT/mTOR signaling pathway. When Tuina therapy was combined with 740 Y-P, a PI3K/AKT/mTOR pathway agonist, PI3K, AKT, and mTOR levels were markedly upregulated. Conversely, co-treatment with LY294002, a PI3K/AKT/mTOR pathway inhibitor resulted in decreased levels of these signaling molecules, although they remained higher than those in the inhibitor-only group. This suggests that Tuina therapy possesses inherent PI3K/AKT/mTOR pathway-activating potential, effectively mitigating the inhibitory effects of LY294002.

Activation of the PI3K/AKT/mTOR signaling pathway regulates chondrocyte autophagy through its downstream biomarkers. Specifically, mTOR pathway activation increases binding affinity to the ULK1 Ser757 phosphorylation site and stabilizes ULK1 protein by inhibiting its proteasomal degradation, thereby suppressing excessive autophagic activity (56). Beclin-1 serves as a critical regulator in autophagosome biogenesis and maturation. LC3 is widely recognized as a specific autophagy marker, undergoing conversion from cytoplasmic LC3-I to membrane-bound LC3-II through phosphatidylethanolamine conjugation on autophagosomal membranes (57, 58). Autophagic flux can be quantitatively assessed by monitoring the LC3-II/LC3-I ratio (with increased LC3-II levels indicating enhanced autophagy). Furthermore, ATG5 and ATG7 function as essential regulatory components in autophagosome formation - their genetic deficiency impairs LC3 lipidation (I→II conversion) and disrupts P62-mediated clearance of ubiquitinated substrates, leading to autophagic flux blockade (59). Our results demonstrated decreased expression of ULK1, Beclin-1, LC3-II/LC3-I ratio, ATG5, and ATG7, along with elevated P62 levels in all experimental groups except the LY294002 group. Compared with the Tuina monotherapy group, the Tuina+LY294002 group had greater reductions in ULK1, Beclin-1, LC3-II/LC3-I ratio, ATG5, and ATG7, and lower P62 levels, whereas the Tuina+740 Y-P group displayed the opposite trend. The TEM findings confirmed that Tuina therapy suppresses chondrocyte autophagy, with its effects being modulated by PI3K/AKT/mTOR pathway inhibitors and agonists. Collectively, these results suggest that Tuina therapy may regulate chondrocyte autophagy in KOA through targeted modulation of the PI3K/AKT/mTOR signaling pathway.

Cartilage degeneration represents the most prominent pathological alteration associated with KOA, with clinical efficacy typically evaluated through combined assessment of cartilage degeneration severity and symptomatic improvement (60–62). In this study, we performed dual morphological evaluation and quantitative analysis of cartilage degeneration using HE staining and Safranin O-Fast Green staining. Morphological observations revealed severe structural damage in the model group, characterized by significant cartilage thinning and extensive chondrocyte necrosis. Post-intervention analyses demonstrated varying degrees of cartilage regeneration across treatment groups, except for the LY294002 group. Notably, the Tuina +740 Y-P combination group exhibited the most substantial therapeutic effects, achieving near-complete restoration of cartilage architecture, chondrocyte regeneration, and cartilage thickness comparable to normal controls. Both monotherapy groups (Tuina alone and 740 Y-P alone) showed moderate cartilage repair. However, the Tuina+LY294002 group displayed minimal improvement, with only slight increases in chondrocyte numbers and partial matrix reorganization, while retaining substantial residual cartilage damage.

Quantitative analyses corroborated these findings, showing that Tuina therapy significantly improved Mankin scores and cartilage area measurements. The therapeutic effects were enhanced when combined with 740 Y-P but diminished with LY294002 co-administration. Behavioral assessments paralleled these staining outcomes: the Tuina+740 Y-P combination had a significantly better effect on increasing PWT and PROM, and reducing KJS, compared with Tuina monotherapy. Conversely, LY294002 administration attenuated the therapeutic effects of Tuina in all parameters measured. Despite this reduced efficacy, Tuina+LY294002 still outperformed both the model group and LY294002 monotherapy group in terms of behavioral improvements. Comprehensive analysis of these multimodal data suggests that Tuina therapy may exert its therapeutic effects on KOA by delaying cartilage degeneration through targeted modulation of autophagy mediated by the PI3K/AKT/mTOR signaling pathway.

Given the limited predictive accuracy observed in animal experiments, we used an SVM model to validate and complement these findings. The SVM was highly accurate, thereby confirming the feasibility and validity of indicator selection within our animal experimental framework. Using the optimal hyperplane separation principle characteristic of the SVM, we predicted both individual and categorical indicator correlations. The analysis revealed strong intra-pathway connectivity within PI3K/AKT/mTOR signaling components, with strong interaction between PI3K and mTOR. PI3K, AKT, and mTOR exhibited significant correlations with autophagy-related indicators, particularly P62 and ATG5. P62 was directly correlated with PI3K/AKT/mTOR signaling components, whereas ATG5 was inversely correlated with PI3K/AKT/mTOR signaling components. Furthermore, six downstream autophagy biomarkers were strongly correlated with the RAL, CAR, and Mankin scores. Categorical correlation analysis demonstrated that cartilage degeneration exhibited the strongest association with behavioral assessments. Additionally, autophagy was significantly correlated with cartilage degeneration and the PI3K/AKT/mTOR pathway. Collectively, these findings suggest a crucial tripartite relationship between PI3K/AKT/mTOR signaling, autophagy regulation, and cartilage degeneration.

We systematically assessed the therapeutic efficacy of various treatments for KOA Through ML analysis. The Tuina+740 Y-P combination had better therapeutic outcomes in both comprehensive and category-specific evaluations than those of the Tuina monotherapy, whereas the Tuina+LY294002 intervention had poorer therapeutic outcomes in both comprehensive and category-specific evaluations than those of the Tuina monotherapy group. Although Tuina monotherapy was more effective than 740 Y-P monotherapy in terms of comprehensive therapeutic outcomes, behavioral performance, and reduced cartilage degeneration, the two modalities had a similar effect on modulating cellular autophagy activity and regulating the PI3K/AKT/mTOR signaling pathway. Comprehensive validation revealed a strong concordance between ML-derived efficacy predictions and the experimental outcomes of the rat study. The ML framework not only confirmed the biological relevance of the selected biomarkers in rat models but also reinforced the validity of the mechanistic conclusions derived from *in vivo* experiments. These findings highlight the utility of ML for both optimizing therapeutic strategies and validating experimental design parameters.

This study has some limitations. First, the intervention effects of Tuina on KOA were evaluated solely through behavioral observations and pathological staining, without imaging evidence. Second, the study did not thoroughly investigate how the physical stimulation generated by Tuina massage translates into the activation of the intracellular PI3K/AKT/mTOR pathway; therefore, the mechano-chemical transduction process was not elucidated. Furthermore, owing to the absence of clinical validation, the conclusions should be regarded as preliminary. Further studies are necessary to confirm the mechanism by which Tuina massage activates the PI3K/AKT/mTOR pathway in treating KOA. In the ML analysis, the limited sample size limited the complexity of the methodological approach. Advanced deep-learning methods such as neural networks, large-scale models, and ensemble learning were not incorporated. These areas require refinement in future research.

## Conclusion

This study demonstrates that Tuina therapy alleviates symptoms in KOA model rats by activating the PI3K/AKT/mTOR signaling pathway, which suppresses excessive autophagy and subsequently reduces cartilage degeneration. These findings provide novel insights into the therapeutic mechanisms of Tuina for KOA management and establish a solid theoretical foundation for its clinical application in KOA treatment.

## Data Availability

The original contributions presented in the study are included in the article/**Supplementary Material**. Further inquiries can be directed to the corresponding author.
