# Supplementary material for: Tuina therapy alleviates knee osteoarthritis by modulating PI3K/AKT/mTOR-mediated autophagy: an integrated machine learning and in vivo rat study
Source: Front Immunol. 2025 Oct 1;16:1635818. doi: 10.3389/fimmu.2025.1635818 (PMC12520871; doi:10.3389/fimmu.2025.1635818)
Supplement: Supplementary file 1 [file DataSheet1.pdf]

Table S1 Instruments and reagents for in vivo experiments.

Name	Company	Catalog Number
Sprague Dawley (SD) male rats	Beijing Vital River Laboratory Animal Technology Co., Ltd, China	SCxK (Jing) 2021–0006
Papain	Beijing Soleibao Biotechnology Co., LTD, China	G8430
L-cysteine	Beijing Soleibao Biotechnology Co., LTD, China	C0012
Sodium pentobarbital	Sigma-Aldrich Corporation, USA	P3761
LY294002	Shanghai Yuanye Biotechnology Co., LTD	S43088
740 Y-P	Beijing Baiolaibo Technology Co., LTD	M00988
Electric von Frey esthesiometer	Stolting lnc, USA	ALMEMO 2450
Dehydrator	Wuhan Junjie Electronics Co., LTD, China	JJ-12J
Embedding machine	Wuhan Junjie Electronics Co., LTD, China	JB-P5
Pathological microtome	Shanghai Leica Instrument Co., LTD, China	RM2016
Frozen table	Wuhan Junjie Electronics Co., LTD, China	JB-L5
Oven	Shanghai Huitai Instrument Manufacturing Co., LTD, China	DHG-9140A
Microwave oven	Galanz microwave electric appliance Co., LTD, China	P70D20TL-P4
Decolorizing shaker	SCILOGEX, USA	CF1524R
Anhydrous ethanol	Sinopharm Group Chemical reagent Co., LTD, China	100092683
Xylene	Sinopharm Group Chemical reagent Co., LTD, China	10023418
Neutral gum	Sinopharm Group Chemical reagent Co., LTD, China	10004160
Positive fluorescence microscope	Nikon, Japan	ECLIPSE
Imaging system	Nikon, Japan	DS-U3
Dewaxing solution	Wuhan Servicebio Biotechnology Co., LTD, China	G1128
PBS buffer	Wuhan Procell Life Technology Co., LTD, China	PB180327
Reverse transcription kit	Beijing Heyi Biological Technology Co., LTD, China	E047-01B
qPCR kit	Beijing Heyi Biological Technology Co., LTD, China	E096-01A
Total RNA extraction reagent	Shanghai Yase Biomedical Technology Co., LTD, China	YY101
Chloroform	Shandong Shuangshuang Chemical Co., LTD, China	GB/T682-2002
Isopropanol	Tianjin Zhiyuan Chemical Reagent Co., LTD, China	1010
4°C centrifuge	Quzhou Xinzhi Biotechnology Co., LTD, China	CF1524R
Ultra-clean table	Suzhou purification Equipment Co., LTD, China	SW-CJ-2D
Nucleic acid quantifier	Thermo Fisher Technologies, USA	Thermo NANODROP 2000
qPCR instrument	Roche, Switzerland	Roche LightCycler480
RIPA	Epizyme, Inc, USA	PC101
Protease inhibitor	Epizyme, Inc, USA	GRF101

Continued Table S1

Name	Company	Catalog Number
Phosphatase inhibitor	Epizyme, Inc, USA	GRF102
PMSF	Beijing Soleibao Biotechnology Co., LTD, China	P0100
BCA protein quantitative kit	Beijing Soleibao Biotechnology Co., LTD, China	PC0020
Nuclear protein extraction kit	Beijing Soleibao Biotechnology Co., LTD, China	R0050
5× Loading buffer	Epizyme, Inc, USA	LT101S
10× electrophoresis buffer	Epizyme, Inc, USA	PS105S
10× transmembrane buffer	Epizyme, Inc, USA	PS109S
Skim milk powder	Epizyme, Inc, USA	PS112
Developing solution	Beijing Soleibao Biotechnology Co., LTD, China	SQ201
10×TBST	Epizyme, Inc, USA	PS103S
Methyl alcohol	Tianjin Hengxing Chemical reagent manufacturing Co., LTD, China	32058
Electron microscope fixative	Wuhan Xavier Biotechnology Co., LTD, China	G1102
Transmission electron microscope	Hitachi, Japan	HT7800
Transmission electron microscope imaging system	Hitachi, Japan	Hitachi TEM system
Ultra microtome	Leica, Germany	Leica UC7
Sklearn.svm tool	Python	3.12.3
GraphPad Prism	GraphPad Software	8.0.2
